# To parachute or not

**DOI:** 10.3332/ecancer.2022.ed125

**Published:** 2022-11-07

**Authors:** Michael L Hicks, Groesbeck P Parham

**Affiliations:** 1 St. Joseph Mercy Oakland, Michigan Cancer Center, 44405 Woodward Ave, Suite 202, Pontiac, MI 48341, USA; 2 University of North Carolina at Chapel Hill, Department of Obstetrics & Gynecology, Chapel Hill, 101 Manning Dr. Chapel Hill, NC 27514, USA; 3 Department of Obstetrics and Gynaecology, University Teaching Hospital – Women and Newborn Hospital, 10101 Nationalist Way Lusaka, Zambia; a https://orcid.org/0000-0002-1819-155X; b https://orcid.org/0000-0001-5922-5990

**Keywords:** noncommunicable diseases, high-income country, low-and middle-income country, Democratic Republic of the Congo, parachuting, resource-constrained settings, cervical cancer, breast cancer

## Abstract

This editorial was prompted by a criticism of our inability to identify all existing local oncologic human resources prior to the initiation of a women’s cancer care platform in the Democratic Republic of the Congo. We discuss the act of parachuting, i.e., intermittent visits by investigators from high-income countries to low-and middle-income countries, its dichotomization (positive and negative), role in bilateral collaborations between high-income and low-and middle-income countries, contributing etiologies and potential harms. Lastly, we highlight our use of parachuting to successfully transfer breast and cervical cancer diagnostic and surgical skills to healthcare providers in a low-income African nation, while simultaneously building clinical infrastructure for women’s cancers. We conclude with recommendations that pertain to the development of better research ecosystems in Africa.

Non-communicable diseases are increasing among populations in low-and middle-income countries (LMIC) [1, 2]. Of particular interest is cancer, the majority of new cases and deaths of which are predicted to occur in the world’s poorest nations over the next two decades [1, 2]. This impending geographic shift in cancer burden has spawned a plethora of international collaborations between organizations and colleagues from LMIC, with the presumed intent of identifying their most critical determinants and developing impactful interventions. Of special interest are cancers of the breast and cervix, given their poor outcomes in resource-constrained settings, juxtaposed against the high cure rates achieved when resources are available and accessible [3–5]. While such partnerships have the potential to yield beneficial outcomes, they themselves can be tainted by misperceptions on the part of the participating parties that inevitably lead to inequities. This is particularly true when programme management involves intermittent visits to the host country by investigators from high-income countries (HIC), often referred to as “parachuting.” The potential shortcomings inherent in this approach have recently been highlighted by researchers and editorial boards of scientific journals [6, 7]. Our editorial is meant to offer a perspective on the matter, as African American oncologists working in Africa.

In the sphere of international research and implementation science, parachuting usually involves HIC collaborators traveling intermittently to a LMIC for purposes of project oversight and management, i.e., monitoring and evaluation, quality control, data collection, adherence to research algorithms, etc. As standard operating procedures, these measures are performed to meet programme requirements and to ensure that activities are proceeding as planned. However, parachuting turns dark (negative parachuting) when visiting HIC investigators attempt to wield their authority by maneuvering and manipulating LMIC personnel and infrastructure, and in some cases even local study participants. This is further accentuated when HIC investigators return to their home base, write and publish manuscripts in prestigious journals, while giving little or insignificant credit or authorship to the respective research collaborators in the LMIC. This form of academic piracy has recently been cited by the journals *ecancermedicascience* and* Lancet*. Both have adopted policies that discourage this practice and instead incentivize the alternative by encouraging the inclusion of LMIC contributors as significant collaborating authors, in accordance with their contributions [6, 7].

Competent professional consultants and well-meaning collaborators from HIC often have genuine altruistic goals and a heartfelt purpose of providing technical assistance in LMIC. However, they frequently lack the necessary training, social awareness and cultural sensitivity to function within socioeconomic and cultural environments outside of the ones to which they are native. These incapacities are frequently exhibited in both the design of their research methodologies and lack of respect and appreciation (overtly or subconsciously) for the expertise and educational background of LMIC collaborators. Details of how to address these matters have been well documented [8].

We recently co-authored a special series published in* ecancermedicalscience* related to oncologic capacity building in the Democratic Republic of Congo (DRC). The activities consisted of quarterly on-site visits to a healthcare facility in Kinshasa, DRC, over a two-year time interval, by a team of U.S. and Zambian specialists in breast and cervical cancer care. The activities resulted in the successful transfer of diagnostic and surgical skills to local Congolese collaborators. In partnership with the healthcare facility where the activities were implemented (Biamba Marie Mutombo Hospital) a new women’s cancer care infrastructure was simultaneously developed that offers life-saving radical surgery for early cervical cancer and breast cancer; oncoplastic and palliative breast surgery; and chemotherapy for cervical and breast cancer [9–12]. Along with compensation for their work, the gain for the HIC collaborators was the satisfaction of expanding women’s oncology services in a country in dire need of such. In the published series noted above, local Congolese collaborators were first authors on 2 of the 4 manuscripts and contributing co-authors on all of them.

Even with the above capacity/infrastructure building and academic successes we were criticized by an African oncologist for the following statement we made in one of the 4 manuscripts [11]: “To our knowledge, there were no clinical or medical oncologists within the country during the time of our training activities and programme implementation.” The criticism was aimed at our lack of acknowledgement of the existence of any certified African medical oncologists in the DRC. We understood and appreciated this heartfelt criticism, and openly welcomed it as constructive, which was the motivation for this editorial. To place the criticism in context, prior to starting the project in the DRC we first conducted a situational assessment, consisting of a stake holders conference in Kinshasa, an internal review of resources provided by the Ministry of Health, interviews with key Informants in the country from both private and public sectors, and a review of the published peer reviewed and gray literature on the subject. The process we followed is clearly stated in the manuscript. Our findings were corroborated by the Congolese authors and co-authors. The fact that we were unable to identify any formally trained and certified medical/clinical oncologists prompted us to include chemotherapy as part of our capacity building activities. Otherwise, we most certainly would have made every effort to invite local clinical oncologists to participate as collaborators, just as we did for gynaecologic and breast surgery. The criticism was characterized “as a lack of recognition of the work of Africans in their environment” and was buttressed by the phrase “how research parachuting must be called out”.

We share the concerns of our critic related to his implicit negative definition of “research parachuting”. Working in Africa over the past two decades with the sole intent of building gynaecologic and breast oncology capacity, we strongly condemn the negative aspects of parachuting. Instead, we embrace the activities exhibited by our constructive approach (positive parachuting) to capacity building, as per our work in the DRC, Malawi and Zambia [9–16]. Our capacity building activities in these countries have always been performed with local experts as peers, lead authors and co-authors [9–16]. Additionally, we have chronicled our work in Africa related to our method of capacity building using the principles of implementation science [17]. Our understanding of the value of the African physician, their training, contributions and sensitivities are thereby documented, and represent the antithesis of this very criticism. As African Americans, based on our own personal and historical experiences in America, we understand the imperialist racist attitude of being undervalued, discredited, excluded, and considered as imposters in the high rent district of global health and academia, while being used as sources of cheap labor to manage clinical research activities in difficult and less than optimal circumstances.

Prevention is the sole solution, which requires education and self-reflection on the part of both HIC and LMIC collaborators. For the investigators from HIC, we suggest that as a preliminary step to engagement they ask themselves the following questions: did I invest time in evaluating the training, expertise and qualifications of the LMIC collaborators; will I treat the local professionals/researchers as peers or as facilitators only; will I collect data and publish manuscripts with no acknowledgement or significant authorship for the LMIC collaborator; will I invite the local LMIC collaborator to be involved from the point of the initial concept and design of the endeavor to publishing the outcomes, with equitable acknowledgment and co-authorship? If the answer to any of these questions is dissentient, we contend that this would constitute the premise of negative parachuting with a biased assumption that collaborators in LMIC have inferior training and knowledge, and are thus unable to conduct first class research and/or provide excellent clinical care. Such assumptions represent the embodiment of imperialistic and colonialist approaches to research, international consultant work and voluntourism.

Perceived but unproven inequalities in qualifications and abilities can beget attitudes that result in inequities, and thereby serve as the basis for negative parachuting in a collaborative relationship. In order to avoid these misperceptions, it is important to establish metrics that allow one to compare the education/training of HIC and LMIC collaborators in specified disciplines. There is a major gap in the literature in this area. In an attempt to address this information deficit, we performed a comparison of a U.S. and African postgraduate training programme in Obstetrics and Gynaecology. The investigation revealed no significant difference in the respective training programmes relative to their clinical and educational metrics and exposure to research [18].

This study sheds light on the issue of equality and equity in training by providing measurable evidence to refute the misperceptions of lack of educational semblance and uniformity, which serve as the basis for negative parachuting. We also contend that in other areas of medicine, where there are research collaborations between HIC and LMIC, equality and equity in training may also be the case, suggesting that this should be explored more vigorously.

We suggest that while appropriately criticizing the negative parachuting practices of collaborators from HIC, and highlighting the above question that must be answered to avert negative parachuting, it is equally important for LMIC investigators and implementers to continue the pursuit of top-notch training; bring this newly acquired knowledge back to Africa, and find ways to sustain and expand it. Strengthening African owned institutions through responsible management and self-sufficiency, with a focus on competency as well as compensation, will lessen donor dependency. Training and certifying one another in African-led institutions will lead to empowerment of Africans to continue performing their own research, and control their collaborations and academic publications.

## Conclusion

We conclude by saying not all parachuting is contemptible. We have referenced a form of positive parachuting that recognizes the work of African collaborators in their research and clinical ecosystems. Additionally, we have identified suggestions for both HIC and LMIC investigators for consideration to employ as a method of prevention of negative parachuting. The premise of an entity/person entering a LMIC from a HIC to collaborate does not have to automatically result in unfavorable sequela for the LMIC collaborator. Rather, it depends on who is in the parachute, their intentions, and how they are managed by their hosts.

## Conflicts of interest

None.

## Funding

None.
